# The effect of donation frequency on donor health in blood donors donating plasma by plasmapheresis: study protocol for a randomized controlled trial

**DOI:** 10.1186/s13063-024-08035-7

**Published:** 2024-03-11

**Authors:** Morten Haugen, Karin Magnussen, Tonje Eiane Aarsland, Lise Sofie Haug Nissen-Meyer, Tor A. Strand

**Affiliations:** 1 Department of Immunology and Transfusion Medicine, Innlandet Hospital Trust, Anders Sandvigs Gate 17, 2609 Lillehammer, Norway; 2 Faculty of Medicine, University of Oslo, Oslo, Norway; 3 Women’s Clinic, Innlandet Hospital Trust, Lillehammer, Norway; 4 Department of Global Public Health and Primary Care, Center of International Health, University of Bergen, Bergen, Norway; 5 Department of Immunology, Oslo University Hospital, Oslo, Norway; 6 Department of Research, Innlandet Hospital Trust, Lillehammer, Norway

**Keywords:** Plasma, Plasma donation, Donor health, Plasmapheresis, Donation frequency

## Abstract

**Background:**

The demand for plasma products is growing, necessitating an increase in plasma collection by plasmapheresis. While the 20th edition of the European Guidelines permits plasma donors in Europe to donate with 96-h donation intervals, the potential short- and long-term consequences of high-frequency plasma donations on donor health remain unknown. This study aims to measure the effect of plasma donation frequency on plasma protein composition, including total serum protein (TSP) and immunoglobulin G (IgG), in Norwegian male blood donors.

**Methods:**

This randomized controlled trial (RCT) included 120 male blood donors who were randomized into two intervention groups and one control group: high-frequency plasma donors (HFPDs) who donated 650 mL of plasma 3 times every 2 weeks, whereas regular-frequency plasma donors (RFPDs) who donated 650 mL of plasma 1 time every 2 weeks. The control group consisted of whole blood donors. The primary outcomes are the concentrations of TSP and IgG.

**Discussion:**

The findings from this study may have implications for recommendations related to donor health and plasma donation frequencies and may contribute to supporting the strategic independence of plasma products in Norway and Europe without compromising donor health.

**Trial registration:**

ClinicalTrials.gov: NCT05179200. Registered December 20th, 2021.

**Supplementary Information:**

The online version contains supplementary material available at 10.1186/s13063-024-08035-7.

## Administrative information

Note: The numbers in curly brackets in this protocol refer to SPIRIT checklist item numbers. The order of the items has been modified to group similar items (see http://www.equator-network.org/reporting-guidelines/spirit-2013-statement-defining-standard-protocol-items-for-clinical-trials/).

**Table:** 

Title {1}	The Effect of Donation Frequency on Donor Health in Blood Donors Donating Plasma by Plasmapheresis: A Randomized Controlled Trial Short title: Donor Health in Repeat Plasma Donors
Trial registration {2a and 2b}.	ClinicalTrials.gov: NCT05179200. Registered December 20th, 2021, https://clinicaltrials.gov/study/NCT05179200 The WHO trial registration data set is included in Supplementary Table 2 in the protocol.
Protocol version {3}	Protocol version: 1.0, July 2023, revised February 2024.
Funding {4}	This study has been granted funding from the Department of Research, Innlandet Hospital Trust, Norway. Reference: 150456 (project number).
Author details {5a}	MH, KM, and TAS conceived the study, contributed to the study design, sample size calculations, and analytical plans. MH obtained the funding. MH, KM, TEA, LSHNM, and TAS have been writing, reviewing, and editing the manuscript. TEA has assisted in the implementation of the collection of dietary data and will contribute to interpreting the dietary data. All authors have read and approved the final manuscript.
Name and contact information for the trial sponsor {5b}	Trial Sponsor: The Department of Research, Innlandet Hospital Trust, Norway Contact name: Tor A. Strand (principal investigator and sponsor representative) Address: Furnesveien 26, 2380 Brumunddal, Norway. Email: tors@me.com
Role of sponsor {5c}	The funding source had no role in the design of this study and will not have any role during its execution, analyses, interpretation of the data, or decision to submit results.
Role of committees {5d}	**Principal investigator and investigators:** Design and conduct the study Preparation of protocol and revisions, agreement of final protocol Recruitment Organizing DMC meetings **Data Monitoring Committee (DMC)/Data and Safety Monitoring Board (DSMB):** Assess the progress of the trial Assess the safety of data Give recommendations to the sponsor, regarding continuation, modification, or termination of the study

## Strengths and limitations of this study

- This is the second RCT that aims to investigate the effect of donation frequency on plasma proteins and donor health.
- This RCT includes 10 repeated measurements of plasma biomarkers over a period of 20 weeks.
- This RCT investigates symptoms of psychological distress in plasma donors.
- The primary outcomes are changes in the concentrations of TSP and IgG in donors during plasma donation at different donation intervals.

## Introduction

### Background and rationale {6a}

The demand for plasma-derived medicinal products (PDMPs), particularly immunoglobulins, is steadily increasing [1, 2]. PDMPs are used for the treatment of a variety of diseases that may be lifelong, and for many PDMPs, no alternative treatment option exists [3, 4].

Human plasma, the main source of PDMPs, can be collected as source plasma through plasmapheresis or as recovered plasma from whole blood donations [3]. In Norway, the reduction in red blood cell donations in the recent years has led to a decline in recovered plasma [1].

The United States is a major contributor, supplying approximately 70% of the global plasma [5], often relying on paid, high-frequency plasma donors, which raises ethical concerns. In Europe, 35–40% of the current PDMP demand depends on plasma collected outside the continent [6]. Europe aims to achieve strategic independence of plasma through voluntary non-remunerated blood donation (VNRBD) to prevent shortages, particularly in immunoglobulins [7]. This necessitates an increase in plasma collection within Europe.

Expanding source plasma collection can be achieved by increasing the donation volume or frequency. Plasma donation volumes and frequencies are governed by national guidelines and vary from country to country. In the USA, plasma donors can donate 880 mL of plasma, including anticoagulant (AC), up to 104 times per year, with a minimum 48-h donation interval [8]. The 20th edition of the European Guidelines allows donors to donate 880 mL of plasma, including AC, up to 33 times annually, with a minimum 96-h donation interval [9]. Norwegian guidelines permit a donation of 650 mL of plasma up to 15 L per year, with a minimum 14-day donation interval [10]. The 20th edition of the European Guidelines recommends adjusting the collection volume and frequency based on the donor’s estimated blood volume (EBV) and immunoglobulin G (IgG) levels [9].

Plasma donation requires sufficient concentrations of total serum protein (TSP) to be measured at least annually and of IgG to be measured annually and in every fifth donation. The most recent European Guidelines from 2023 [11] recommend a minimum donation interval of 1 week. Current studies on donor health and plasma donation frequency primarily consist of observational studies examining biochemical data, with limited controlled experimental studies [12, 13]. The Council of Europe emphasizes the need for further short- and long-term prospective studies regarding donor health [9].

Previous studies have reported significantly reduced levels of TSP and/or immunoglobulins during plasmapheresis of 500–1500 mL per week [12, 14–17]. However, two similar studies reported no significant decrease in serum protein levels [18, 19]. Another study involving less extensive plasmapheresis of 550 mL every 2–3 weeks over 10 years revealed that protein levels remained within normal ranges [20].

A recent cross-sectional study of 483 participants, including 100 non-donors, revealed significantly lower TSP, albumin, and IgG levels in donors undergoing plasmapheresis at different frequencies than in non-donors. This study confirmed the safety of long-term intensive donor plasmapheresis, with up to 45 L of plasma per year, in terms of cellular immunity, red cell and iron metabolism, and cardiovascular risk markers [21]. However, another cross-sectional study [22], using pooled plasma samples from different countries with varying donation frequencies and volumes, revealed that high-frequency and high-volume plasma donations limited the ability of plasma proteins, especially immunoglobulins, to return to normal physiologic levels. Increased levels of C-reactive protein (CRP) were observed in plasma from high-frequency plasma donors, suggesting potential activation of inflammatory processes, which may impact donor health. This study also revealed severe decreases in IgG1, IgG2, and IgG4 in paid high-frequency plasma donors compared to unpaid blood donors and low-frequency plasma donors.

IgG subclasses 1–4 were investigated in a study of 403 regular plasmapheresis donors [23], with values not significantly different from those of non-donors. However, the concentration of IgG4 was significantly reduced between the two subsequent donations, and the percentage of donors with values below the normal ranges of IgG subclasses 1–4 increased during the donations.

A prospective multicenter study, the SIPLA study [24], focusing on the safety of long-term intensive plasmapheresis included 3783 experienced plasmapheresis donors who were switched from a moderate to an intensive plasmapheresis regime over 3 years (750 mL per session and at least once a week, or 850 mL voluntarily). The results indicated a significant reduction in IgG and TSP levels, leading to a 16% exclusion rate due to low IgG (< 5.8 g/L), TSP, or hemoglobin (Hb) levels. However, the high total dropout rate of 75% may bias the observed associations.

The first randomized controlled trial (RCT) investigating the effect of plasma donation frequency on donor health [13] aimed to collect data on biochemical markers, physiological and exercise-related parameters, and adverse events (AEs) in donors donating plasma once per month to twice per week. The results from this trial revealed significant reductions in the levels of serum albumin, IgG, IgA, and IgM in donors who donated plasma twice per week compared to those in the control group.

A recent scoping review on donor health in plasma donors [25] identified existing evidence and evidence gaps and concluded that additional experimental studies on the health effects of different donation frequencies are needed to establish a safe upper limit for plasma donation frequency.

In this RCT, we aimed to estimate the effect of plasma donation frequency on blood donor health by comparing Norwegian male blood donors donating 650 mL of plasma excluding AC by plasmapheresis. Blood donors were randomized into two plasma donation groups: high-frequency plasma donors (HFPDs), who donate plasma 3 times every 2 weeks, and regular-frequency plasma donors (RFPDs), who donate plasma 1 time every 2 weeks. Donors in the control group donated whole blood at 3-month intervals. Repeated measurements of the concentrations of TSP and IgG, along with other plasma proteins, lipids, and hematological biomarkers, were collected. Information about nutritional status and symptoms reflecting psychological distress in donors was also collected.

This study is important for ensuring the health of high-frequency plasma donors, and to the best of our knowledge, this will be the second RCT examining donor health related to plasma donation frequency. The findings from the study may contribute to increased and safe plasma collection, ensuring European strategic independence of plasma without compromising donor health.

### Objectives {7}

#### Research hypothesis

High-frequency plasma donation of 650 mL of plasma 3 times every 2 weeks is non-inferior to both plasma donation of 650 mL of plasma 1 time every 2 weeks and whole blood donation in terms of donor health based on differences in the concentrations of TSP, IgG, and various other specific plasma proteins.

#### Primary objective

1. To compare the concentrations of TSP (g/L) and IgG (g/L) at baseline, during a 16-week donation period, and after a 4-week follow-up period between the HFPDs, RFPDs, and controls.

#### Secondary objectives

1. To compare the concentrations of other plasma proteins, including IgG subclasses, lipids, hematological biomarkers, and biomarkers reflecting nutritional status, inflammation, and other processes related to health and disease between the HFPDs, RFPDs, and controls.
2. To compare symptoms of psychological distress before and after the donation period between the HFPDs, RFPDs, and controls using the Hopkins Symptoms Checklist 25 (HSCL-25).
3. To compare for habitual diet between the HFPDs, RFPDs, and controls, using a self-administered food frequency questionnaire (FFQ).
4. To compare the dropout rate and reasons for dropout between the HFPDs, RFPDs, and controls.
5. To compare the AEs, and evaluate their relationship to plasma/blood donation, between HFPDs, RFPDs and controls.

### Trial design {8}

This non-inferiority RCT included 120 male blood donors randomized at a 1:1:1 ratio. The study comprised two intervention groups that donate 650 mL of plasma excluding AC 3 times every 2 weeks and 1 time every 2, respectively, and one control group consisting of regular blood donors donating 450 mL of whole blood every 3 months. Participants were followed over a 16-week intervention period and a 4-week follow-up period.

## Methods: participants, interventions, and outcomes

### Study setting {9}

The study was performed at the Blood Centre, Innlandet Hospital Trust (IHT), Norway, at four different donation sites: Elverum, Hamar, and Lillehammer (all since 2022) and Gjøvik (since 2023). These donation sites serve blood donors from the small cities/rural areas where the donation sites are situated and the surrounding rural areas. All donation sites adhere to established routines and have trained staff specifically equipped for plasma donations.

### Eligibility criteria {10}

#### Inclusion criteria

1. Established male blood donors aged between 18 and 64 years who met the eligibility criteria for both whole blood and plasma donation by plasmapheresis according to the current guidelines [9, 10].
2. Donors must have a history of at least one prior plasma donation.
3. Concentrations of Hb ≥ 13.5 g/dL, IgG ≥ 6.0 g/L, and TSP ≥ 60 g/L.
4. The estimated blood volume (EBV) determined by the ISCH formula [26] must be at least 4500 mL.

#### Exclusion criteria

1. History of repeated measurements (> 2) of hematocrit > 50% before enrollment in the trial.

All participants must undergo at least 2 months of quarantine after any type of donation before being included in the study. This allows plasma proteins to adjust to physiologic levels.

##### Rationale

The concentration limits for Hb, IgG, and TSP are established criteria and safety markers for blood donors donating either whole blood or plasma, according to European and National guidelines [9, 10]. The exclusion criterion related to hematocrit levels is implemented to mitigate potential complications during the plasmapheresis procedure, particularly in the context of prolonged donation times. Participation is gender-restricted (male sex) to reduce the variability among the participants and to standardize the plasma donation volume to 720 mL, including AC, thereby enhancing the internal validity of the study.

Although the study population will be representative of most Norwegian Caucasian male plasma donors, it is essential to note that the findings may not be generalizable to female blood donors or individuals with different ethnic backgrounds.

### Who will take informed consent? {26a}

Trained technicians provided oral and written information to the blood donors regarding the study. The blood donors had the opportunity to pose questions to the researchers and were afforded time to reflect upon the information before providing written consent. The technicians then collected written consent from the blood donors who were willing to participate. All the information sheets and consent forms used were available in the Norwegian language.

### Additional consent provisions for collection and use of participant data and biological specimens {26b}

The written consent form included information about the collection of plasma and blood cells for biobanking. The collected material will be stored until the end of the project period on August 1, 2031, and may be utilized for ancillary studies within this specified period.

## Interventions

### Explanation for the choice of comparators {6b}

In this trial, we chose active donors who donated 450 mL of whole blood with a minimum donation interval of 3 months as comparators. Consequently, the blood donors in the control group donated a total volume of up to 500 mL of plasma throughout the trial. Given that blood donors represent a distinct cohort of healthy individuals, it is imperative to mitigate potential healthy donor bias by selecting active donors as a control group [27].

### Intervention description {11a}

Written informed consent was obtained from all participants prior to randomization and the initiation of any intervention. The donors who provided written informed consent were then randomized into two intervention groups, both of which underwent plasma donation for 16 weeks:

1. HFPDs donated 650 mL of plasma, excluding AC, by plasmapheresis 3 times every 2 weeks, for a total of 24 donations. The donation frequency adhered to the European Guidelines, necessitating at least 96-h donation intervals between donations [9].
2. RFPDs donated 650 mL of plasma, excluding AC, by plasmapheresis 1 time every 2 weeks, for a total of 8 donations. The donation frequency adhered to national guidelines [10].

The plasmapheresis procedure was performed using the Aurora Plasmapheresis machine, Fresenius Kabi. Sodium citrate (4%, 10.0 g/250 mL) was added as AC at a whole blood-to-anticoagulant ratio (ACR) of 100:6. The standardized plasma donation volume was set at 720 mL, including AC, in each procedure, adhering to the maximum recommended plasma donation volume according to the national guidelines. This corresponds to approximately 650 mL of plasma, excluding AC, assuming a hematocrit of 44% and an ACR of 100:6. Fluid replacement was not administered during the procedure. A donation of 650 mL of plasma necessitates a minimum estimated blood volume of 4150 mL, according to the 20th edition of the European guidelines [9]. Considering blood sampling of up to a maximum of 37.5 mL and incorporating a safety margin of approximately 4%, the inclusion criterion was an EBV of 4500 mL.

### Criteria for discontinuing or modifying allocated interventions {11b}

AEs graded 1–2 and medical issues were individually assessed, and they could result in a temporary deferral from the intervention or permanent discontinuation. Examples of such issues include hematoma, infiltration, or citrate reactions. Grade 3–5 AEs lead to permanent discontinuation of participation.

Participants could be temporarily deferred from intervention due to low Hb levels (< 13.5 or < 13.0 g/dL for blood and plasma donation, respectively), low IgG levels (< 6.0 g/L), or low TSP levels (< 60 g/L). If the concentrations of Hb, IgG, and/or TSP fell below these thresholds in two subsequent donations, a deferral for 2 weeks was implemented, and a reassessment was conducted at the next donation. If the new measurements indicated concentrations above the thresholds, the donor could resume donations. If the new measurements showed concentrations below the thresholds, the donor was monitored with blood samples only.

Incomplete plasmapheresis procedures, including aborted re-infusion of red cells, were not considered successful interventions, and could lead to a temporary discontinuation of the intervention, depending on the blood volume that is not re-infused.

### Strategies to improve adherence to interventions {11c}

Strategies to improve adherence to intervention protocols included providing participants with information about their laboratory results and ensuring follow-up. In Norway, blood and plasma donations are based on the principle of VNRBD. Participants in the trial received only symbolic blood donor gifts for each visit. This approach is intended to reinforce the voluntary and altruistic nature of blood and plasma donation, promoting continued participation based on the goodwill of the donors rather than monetary incentives.

### Relevant concomitant care permitted or prohibited during the trial {11d}

Throughout the trial duration, donors were prohibited from donating blood or plasma beyond what is described in this protocol. There was no requirement for participants to alter their lifestyle or dietary habits during the study. Participants were informed about the importance of maintaining adequate hydration before each plasma donation.

Blood and plasma donors could be advised to take iron supplements if necessary, following an evaluation based on Hb and ferritin levels in accordance with local procedures. In the event of citrate reactions during plasma donation, donors would be provided with milk or oral calcium.

Participants were encouraged to continue taking medications for any existing medical conditions as prescribed. Eligibility was assessed on the basis of adherence to the inclusion criteria specified in the study protocol.

### Provisions for post-trial care {30}

Participants will receive compensation for healthcare needs arising from any intervention-related harm occurring during and after the trial. Additionally, participants could resume regular donation after the conclusion of the trial.

### Outcomes {12}

#### Primary outcome measures

1. Change from baseline of the mean TSP and IgG concentrations (g/L) until the endpoint of the last donation.

#### Secondary outcome measures

1. Change from baseline in the mean concentrations of plasma proteins, including IgG subclasses, and other biomarkers (Supplementary Table 1) until the endpoint of the last donation.
2. The change in the mean overall score and score for each item of the HSCL-25 from baseline until after the donation period, including the proportion of participants with an overall score ≥ 1.75 after the donation period.
3. The number of dropouts and reasons for dropout during the study period.
4. The sum of AEs during the study period.
5. Estimated daily intake of various nutrients (e.g., protein, fat, carbohydrates, vitamin D, folate, and iron) in energy or mass equivalents obtained from the FFQ.

#### Rationale for outcomes

This study aimed to measure a comprehensive set of health outcomes, including biomarkers and mental health. With 10 repeated measurements for biomarker outcomes, concentration trajectories will be explored. Some of the variables may serve as both independent and dependent variables in the planned analyses.

The primary outcomes, TSP and IgG, serve as biomarkers of safety in plasmapheresis donors, according to established guidelines [9–11]. Immunoglobulins and IgG subclasses are indicators of a compromised immune system and potential infection risk. The measurement of various plasma proteins (albumin, transferrin, immunoglobulins, CRP, and lipoproteins) will provide knowledge on the fractions of proteins that high-frequency plasma donors might risk being deficient in.

The reduction and recovery of some of the biomarker concentrations should be interpreted in relation to dietary intake; therefore, daily energy and nutrient intake will be estimated using an FFQ at the end of the study period.

While previous observational studies have investigated the effect of plasma donation on biomarkers associated with cardiovascular disease in blood donors [21, 28, 29], this study contributes to the knowledge gap by investigating the effect of plasma donation frequency on cardiovascular markers in an RCT. We will also include liporotein (a) as a novel marker of cardiovascular disease [30].

Psychological distress in high-frequency donors will be investigated. Prior research has shown concerns among plasmapheresis donors about potential negative effects on their health or well-being [31], and this study aimed to explore the psychological effects in high-frequency plasma donors, which, to our knowledge, has not been extensively investigated before.

### Participant timeline {13}

The participant timeline is shown in Table 1. Every participant screened for eligibility was assigned a screening ID. The participants who provided written informed consent were randomized as closely as possible before the intervention period. The questionnaires were completed approximately 1 week prior to the first donation and 1 week after the last donation.

**Table 1:** Participant timeline

	Enrollment	Allocation	Post allocation								Follow-up	
Timepoint (week)	− 1	0	1–2	3–4	5–6	7–8	9–10	11–12	13–14	15–16	17–18	19–20
Enrollment:												
Eligibility screen	x											
Informed consent	x											
Allocation		x										
Interventions:												
HFPDs: Plasmapheresis			xxx	xxx	xxx	xxx	xxx	xxx	xxx	xxx		
RFPDs: Plasmapheresis			x	x	x	x	x	x	x	x		
Controls: Blood donation			x							x		
Assessments:												
Baseline variables: Outcome variables:	x	x										
Blood samples			x	x	x	x	x	x	x	x	x	x
FFQ											x	
HSCL-25		x									x	

*FFQ* food frequency questionnaire, *HSCL-25* Hopkins Symptom Checklist-25

### Sample size {14}

The sample size calculation was conducted using an online platform (Sealed Envelope) [32], considering an anticipated dropout rate of up to 20%. The standard deviations of TSP and IgG were derived from records from male blood donors at the Blood Center at IHT and were 4 and 2 g/L for TSP and IgG, respectively.

Based on these assumptions, a sample size of 40 participants in each group was determined. In other words, if there was truly no difference between the standard and experimental interventions, 32 participants per group were required to have 80% confidence that the lower limit of a one-sided 95% confidence interval (or equivalently a 90% two-sided confidence interval) would be above the non-inferiority limit of − 3.0 (TSP) or − 1.5 (IgG). The chosen non-inferiority limits were discussed among the clinicians in the study team, and with these limits, higher and more clinically relevant differences can also be detected.

Considering the restricted enrollment of only males in this study population, it is expected that the variability of the outcomes will be lower than initially anticipated. As a result, the precision of the effect measure estimates is anticipated to be greater than that indicated by these calculations.

### Recruitment {15}

Established blood donors who met the eligibility criteria were actively recruited for the trial. Information about the trial was disseminated by the Norwegian Red Cross. Participants were recruited until 40 participants were randomized into each group.

## Assignment of interventions: allocation

### Sequence generation {16a}

A randomized allocation sequence was generated using Stata/SE 16.1 (StataCorp, College Station, TX, USA). The sequence was generated by an external investigator who was not otherwise involved in the study.

### Concealment mechanism {16b}

The randomization was conducted through block randomization at a 1:1:1 ratio, with a variable block size (3 or 6) and stratification based on the donation site to ensure an equal distribution of participants in each group at every site. Allocation concealment was maintained because the digital randomization list was solely accessible to the external investigator and remained undisclosed to the investigators responsible for participant recruitment.

### Implementation {16c}

The process of sequence generation and allocation concealment was distinct from the process related to the implementation of study groups. The external investigator retained control of the list that links the study ID number to the randomization code. Following enrollment, participants were sequentially assigned a study ID number. Subsequently, the external investigator was responsible for allocating participants to one of the intervention groups or the control group based on their respective study ID number.

## Assignment of interventions: blinding

### Who will be blinded {17a}

Participants, care providers, outcome assessors, and statistical analyses were not blinded in this study. This lack of blinding should be considered in the interpretation and discussion of the study results, as it introduces the potential for bias.

### Procedure for unblinding if needed {17b}

Not applicable.

## Data collection and management

### Plans for assessment and collection of outcomes {18a}

Baseline data, including participant age, blood type, and donation history, were collected from the Blood Centre’s data system (LabCraft AS, Oslo, Norway, version 6.8.0) [7]. A questionnaire was utilized at baseline with additional background information about the donor, such as education, occupation, and civil status, to provide a comprehensive description of the study population.

Furthermore, a structured form was used for each participant to record information such as height and weight for estimating blood volume. This form also captured attendance, procedures conducted, and any adverse events (AEs) or complications during each procedure.

At baseline, the participants were requested to complete the HSCL-25. During the follow-up period after the donation period, the participants were asked to complete the FFQ. They were also asked about whether and how often they were willing to donate plasma in the future, and the HSCL-25 was repeated to assess any changes in psychological distress.

#### Laboratory procedures

All blood samples were collected every 2 weeks, totaling 10 samples from each participant throughout the study, as detailed in Supplementary Table 1. These samples were collected prior to the initiation of plasma or blood donation and were not influenced by the sodium citrate used in the plasmapheresis kit or anticoagulant in the blood bags.

The baseline blood sample (sample 1) was collected before the donation period began, and samples 2–8 were collected during the donation period. During the follow-up period, blood samples 9 and 10 were collected 2 and 4 weeks after the last donation, respectively. Due to a longer donation period of 1 week for the HFPDs, samples 8–10 for the HFPDs were collected 1 week later than those for the RFPDs and controls. This is acknowledged as a limitation of the study design, which could overestimate the effect in the HFPDs. Adjustments will be made in the statistical analyses, if necessary, and this aspect will be discussed as a limitation in the publications.

All tubes were centrifuged at 2200 × *g* at room temperature for 10 min. The blood samples were promptly analyzed at the laboratory at IHT according to local procedures, utilizing established reference ranges for men. Any abnormal test results were carefully evaluated, and participants were referred to their general practitioner for further evaluation. If deficiencies in vitamin D (< 50 nmol/L), folate, or vitamin B12 were identified, appropriate investigations were conducted, and the participants received general dietary advice along with recommendations for relevant supplementation.

#### Biobank

For biobank sampling, 6 mL of EDTA blood was collected. Until further processing and freezing, the sample was stored at 4 °C. Within 3 h after collection, the tube was centrifuged at room temperature. The plasma was homogenized and divided into three aliquots of 500 µL each. The buffy coat and red cells were collected into one aliquot of 500 µl from the blood sample at baseline and at the time of the last donation (numbers 1 and 8), exclusively. Thereafter, all biobank samples were stored at − 40 °C. Every 6 months, biobank samples were collected from the donation sites and transferred to − 80 °C until analysis.

#### Collection of data on psychological distress

The HSCL-25 is a screening tool designed to measure symptoms of anxiety and depression [33]. With respect to 25 questions, with 10 focused on anxiety and 15 on depression, respondents rated each statement on a 4-point scale from “not at all” (1) to “extremely” (4). The mean of the scores, ranging from 1–4, is strongly correlated with overall psychological distress [34].

In previous studies, a score ≥ 1.75 has been suggested as a clinical cut-off [34–37] and has been employed to examine trends over time by comparing changes in scores [38]. In this study, a Norwegian translation of HSCL-25 was used to measure psychological distress experienced in the preceding 2 weeks. The HSCL-25 was administered at baseline and repeated after the donation period to investigate any changes in both the total mean score and the mean score for each question.

#### Collection of dietary data

Dietary data were collected using a validated self-administered semi-quantitative FFQ developed at the Department of Nutrition, University of Oslo [39]. This FFQ is designed to capture the habitual food intake of Norwegian adults in the preceding 3 months, and the participants provided information on the frequency of consumption and typical portion sizes of various foods.

To calculate the intake of energy and nutrients, the provided dietary information was processed using the food composition database and calculation system “Kostberegningssystem” (KBS) from the University of Oslo. The participants received access to the FFQ through a direct link sent via SMS.

The concentrations of biomarkers reflecting nutritional status were then compared to the calculated intake of the corresponding nutrients, which allowed for an assessment of the combined influence of both plasma donations and dietary intake on nutritional status.

### Plans to promote participant retention and complete follow-up {18b}

To promote participant retention and complete follow-up, participants maintained regular communication with the technicians at each collection site. Generally, blood donors exhibit strong motivation and dedication as research participants. Participants reserved the right to withdraw from the study at any time without being obligated to provide a reason. However, if the reason for withdrawal was not apparent, the participants were asked to share their reason for the withdrawal. Reasons for dropout were systematically recorded and categorized, and medical reasons related or unrelated to plasma or blood donations, as well as socioeconomic factors, were distinguished.

### Data management {19}

All the data were documented in an Excel database designed for the study. The data are securely stored on dedicated research servers at IHT, adhering strictly to national regulations for information security. All study-related documents, including consent forms and various data collection forms, are stored and archived for 5 years following the conclusion of the project period, extending until 2036.

Access to data is restricted to two designated individuals, namely, one researcher and one project technician.

### Confidentiality {27}

Personal information about the screened and enrolled participants is securely stored on dedicated research servers. The list of participants, their identities, and corresponding consent forms are stored separately from the data. These hard copies are secured within locked file cabinets at IHT.

### Plans for collection, laboratory evaluation, and storage of biological specimens for genetic or molecular analysis in this trial/future use {33}

The plasma and cells stored in the biobank have the potential for use in future analyses of biomarkers reflecting processes related to health and disease, nutritional status, and inflammation. The biobank samples are kept during the project period and may be used for analysis during this period.

## Statistical methods

### Statistical methods for primary and secondary outcomes {20a}

Most of the data analysis will be conducted using Stata (Stat Corp, College Station, TX, USA) statistical software package. All participants who were randomized will be included in the analyses if the relevant outcome variables were collected.

The primary outcomes are continuous and expected to be normally distributed. We will use the concentrations of the biomarkers from blood samples at baseline (sample number 1) and at the endpoint (sample number 8) to calculate the mean difference between the baseline and the last donation in each group. This difference will be compared between the groups in addition to the group means at each time point.

For secondary outcomes, the frequencies of AEs between the groups will be compared. The time until AEs and/or dropouts between the groups will be compared using Cox proportional hazards models. The change in the total HSCL-25 score from baseline until after the intervention will be compared by linear regression. Proportions scoring above the cut-off value will be compared using logistic regression. The change in the HSCL-25 score for individual questions will also be examined.

Finally, in generalized linear mixed effects models, we will estimate the interaction effect between time and group identity on the concentrations of the different biomarkers, including the primary outcomes. These analyses will incorporate the recovery of plasma proteins two and 4 weeks after the last donation (samples 9 and 10) to model the trajectories of the biomarker concentrations across the different groups. Detailed information on the statistical methods used will be published in the statistical analysis plans for each paper before commencing the data analyses. These statistical analysis plans will be published at ClinicalTrials.gov.

### Interim analyses {21b}

Interim analyses were not conducted since the intervention is a well-established procedure, and serious harm is not anticipated. The participants were closely monitored during the entire study period, as described in “Criteria for discontinuing or modifying allocated interventions {11b}”. The data safety monitoring board (DSMB) promptly evaluated potentially serious adverse events (SAEs), in addition to dropout rates, and AEs every 6 months.

### Methods for additional analyses (e.g., subgroup analyses) {20b}

The analyses will be conducted separately within subgroups based on baseline IgG concentrations (6.0–7.9, 8.0–9.9, and ≥ 10.0 g/L). Different donation intervals are recommended according to the European Guidelines, suggesting 2 weeks for IgG concentrations between 6.0 and 8.0 g/L, 1 week for IgG concentrations between 8.0 and 10.0 g/L, and more frequent donations for IgG concentrations > 10.0 g/L. These subgroup analyses could impact and reduce the statistical power, as the primary outcome calculations require 40 participants for adequate statistical power.

### Methods in analysis to handle protocol non-adherence and any statistical methods to handle missing data {20c}

The data will be analyzed with both intention-to-treat and per-protocol analyses to mitigate potential bias arising from the healthy donor effect [40, 41]. The intention-to-treat population encompasses all randomized participants, excluding permanent dropouts, irrespective of the number of completed donations. The per-protocol population will include participants who completed at least 87.5% of the scheduled donations in the intervention groups (at least 21 in the HFPDs and 7 in the RFPDs). This allows HFPD absence from three visits and RFPDs from one visit, which contributes to reducing selection bias in per-protocol populations.

Missing data will be ignored. Efforts will be made to collect data, such as blood samples, even if the donor is ineligible to donate at a particular visit for other reasons, aiming to minimize the extent of missing data.

### Plans to give access to the full protocol, participant-level data, and statistical code {31c}

The full study report, the anonymized data set, and the statistical code will be made available from the corresponding author upon reasonable request after the main results have been published and as long as it corresponds with the local regulations for data.

## Oversight and monitoring

### Composition of the coordinating center and trial steering committee {5d}

A coordinating center or a trial steering committee was not designated, as the trial was conducted at a single blood center. The principal investigator, TAS, was coordinating the trial, with responsibilities outlined for the other researchers as listed in the section “Authors’ contributions {31b}”.

### Composition of the data monitoring committee, its role and reporting structure {21a}

A DSMB/Data Monitoring Committee (DMC) was established and consisted of a specialist in immunology and transfusion medicine, an anesthesiologist, and a biostatistician. The DMC operated independently from the study sponsor and had no competing interests. The responsibilities of the DSMB were outlined in a charter (included in the supplemental file), which included the study-stopping rules and guidelines for reporting SAEs and AEs.

### Adverse event reporting and harms {22}

Plasma and blood donation may induce minor side effects, which are typically mild, including vasovagal reactions, iron depletion, dehydration, arm pain, and citrate reactions. Major side effects are rare. Any harm arising after the donor has left the donation site is uncommon. The frequency of some side effects might increase with increasing donation frequency, but high-frequency donors also tend to gain experience and establish relationships with donation site staff.

Participants were monitored during each donation and for approximately 10 min afterward, as most adverse events are likely to occur within this timeframe. The technician who conducted the procedure recorded any AEs, and if necessary, a medical doctor at the donation site was contacted. Prior to each donation, the donors were interviewed and asked about any health issues since their last visit. Data on AEs between donations were collected on a non-systematically basis through this spontaneous reporting.

All harms and AEs were documented and reported in the publications, categorized according to the “Standards for Surveillance of Complications related to blood donations,” consistent with MedDRA standardized terms [42]. The severity and imputability of complications were evaluated using the “Severity Grading tool for Blood Donor Adverse Events” [43] from 1 to 5, based on the Common Terminology Criteria for Adverse Events (CTCAE) [44]. AE grade 3–5 resulted in permanent discontinuation from the study. The investigator assessed the causal relationship to plasmapheresis as “definite or certain,” “probable or likely,” “possible,” “unlikely or doubtful,” or “excluded” [43]*.*

All SAEs were reported to the DSMB within 24 h of awareness of the event. The investigator and the DSMB assessed the relation of the SAEs to plasmapheresis or blood donation based on the report of the event. Regular reporting of AEs occurred at scheduled meetings every 6 months and after follow-up of the first 60 participants.

### Frequency and plans for auditing trial conduct {23}

Auditing was not conducted in this trial.

### Plans for communicating important protocol amendments to relevant parties (e.g., trial participants, ethical committees) {25}

Important protocol modifications were communicated to the research ethics committee and, if necessary, to the trial participants.

### Dissemination plans {31a}

The findings from this study will be published in international peer-reviewed journals and presented at international conferences. The trial participants will be kept informed about the trial’s progress through updates on the web pages of IHT.

## Discussion

This RCT investigates the effect of plasma donation frequency on plasma proteins and other health indicators in Norwegian male blood donors donating plasma. This study compares the plasma donation frequencies 3 times every 2 weeks (in accordance with the 20th edition of the European guidelines [9]) with 1 time every 2 weeks. The primary outcome is the change in the concentrations of TSP and IgG from baseline to the last donation over a 16-week period. This study is the second RCT on the influence of donation frequency on donor health.

The findings from this RCT are expected to provide insights into donor safety among high-frequency plasma donors, focusing on plasma protein composition. The results may offer valuable evidence for recommendations and guidelines on plasma donation frequency, a critical aspect given the rising demand for plasma products and the need to enhance plasma collection without compromising donor health.

Despite these contributions, this RCT has several limitations. The 16-week intervention period, along with the 4-week follow-up after the donations, may be insufficient for investigating the prevalence or risk of infections in repeat plasma donors. Additionally, the inclusion of only male donors might limit the generalizability of the findings to female plasma donors, emphasizing the importance of future research on female donors with adjusted donation volumes. The fixed donation volume of 650 mL of plasma excluding AC may affect the influence of plasma donations depending on the donor’s EBV. Therefore, long-term experimental studies on clinically relevant outcomes in male and female blood donors, including various donation frequencies and volumes adjusted to EBV, are needed in the future.

## Trial status

Protocol version: 1.0, July 2023, revised February 2024. The inclusion of participants started on January 3, 2022, and data collection is expected to be completed by the end of June 2024. As of February 2024, we have enrolled 120 participants. Project period: August 2 2021–August 1 2031.

### Supplementary Information

**Additional file 1: Supplementary Table 1. **Laboratory analyses and methods. **Supplementary Table 2.** WHO Trial Registration Data Set.

**Additional file 2. **Data and Safety Monitoring Board (DSMB) Charter. 

## Data Availability

The data sets from the current study are available from the corresponding author upon reasonable request after the main results have been published, as long as it corresponds with the local regulations for data sharing.

## Abbreviations

AC: Anticoagulant
ACR: Anticoagulant ratio
AEs: Adverse events
CRP: C-reactive protein
DSMB: Data safety monitoring board
DMC: Data Monitoring Committee
EBV: Estimated blood volume
FFQ: Food frequency questionnaire
Hb: Hemoglobin
HFPDs: High-frequency plasma donors
HSCL-25: Hopkins Symptoms Checklist 25
IgG: Immunoglobulin G
IHT: Innlandet Hospital Trust
KBS: “Kostberegningssystem”
LDL: Low-density lipoprotein
PDMPs: Plasma-derived medicinal products
RFPDs: Regular-frequency plasma donors
SAEs: Serious adverse events
TSP: Total serum protein
VNRBD: Voluntary non-remunerated blood donation
