# Supplementary material for: The effect of donation frequency on donor health in blood donors donating plasma by plasmapheresis: study protocol for a randomized controlled trial
Source: Trials. 2024 Mar 11;25:175. doi: 10.1186/s13063-024-08035-7 (PMC10926559; doi:10.1186/s13063-024-08035-7)
Supplement: Supplementary file 1 — Additional file 1: Supplementary Table 1. Laboratory analyses and methods. Supplementary Table 2. WHO Trial Registration Data Set. [file 13063_2024_8035_MOESM1_ESM.docx]

# Supplementary

**Supplementary Table 1:** Laboratory analyses and methods.

| **Tube** | **Analysis, measure unit (reference range)** | **Method** |
| --- | --- | --- |
| EDTA 3 mL | Hb, g/dL (13.4–17.0) | Absorption photometry 540 nm |
|  | Thrombocytes, x10^9/L (145–390) | Optical scatter/MAPSS technology |
|  | Hematocrit, L/L (0.42–0.52) | Calculated: (RBC x MCV)/1000 |
|  | Red blood cells (RBC), x10^12/L (4.3–5.7) | Optical scatter/MAPSS technology and fluorescence |
|  | Middle corpuscular volume (MCV), fL (86–102) | Optical calculated from the mean size of RBC (RDW) |
|  | Middle corpuscular hemoglobin (MCH), pg (27–33) | Calculation: Hb/RBC x 10 |
|  | Middle corpuscular hemoglobin concentration (MCHC), g/dL (30–34) | Calculation: Hb/HCT x 100 |
|  | Leukocytes, x10^9/L (3.5–10.0) | Optical scatter/MAPSS technology and fluorescence |
|  | Differential count:   - Neutrophils, x10^9/L (2.0–7.5) - Lymphocytes, x10^9/L (1.5-4.0) - Monocytes, x10^9/L (0.2–0.8) - Eosinophils, x10^9/L (0.0–0.4) - Basophils, x10^9/L (0.0–0.1) | Optical scatter/MAPSS technology and fluorescence |
| Serum 3.5 mL | Immunoglobulin G, g/L (6.1–14.9) | Nephelometry |
|  | IgG subclasses:  IgG1, g/L (4.05–10.11)  IgG2, g/L (1.69–7.86)  IgG3, g/L (0.110–0.850)  IgG4, g/L (0.030–2.010) | Nephelometry |
|  | Immunoglobulin M, g/L (0.4–2.1) | Nephelometry |
|  | Immunoglobulin A, g/L (0.7–3.7) | Nephelometry |
| Serum 3.5 mL | Lipoprotein (a), mg/L (0–300)  Apo lipoprotein B-100 (ApoB), g/L (0.7–1.6) | Immunoturbidimetry (C-module) |
| Serum or lithium-heparin plasma (3.5 mL) | Total serum protein, g/L (62–78) | Biuret method (C-module) |
|  | Ferritin, µmol/L (30–400) | Immunoassay (I-module) |
|  | Transferrin, g/L (1.9–3.3) | Immunoturbidimetry (C-module) |
|  | C-reactive protein (CRP), mg/L (0–5) | Immunoturbidimetry (C-module) |
|  | Creatinine, µmol/L (60–105) | Enzymatic assay (C-module) |
|  | Vitamin B12, pmol/L (140–600) | 2-step immunoassay (I-module) |
|  | Folate, nmol/L (7–40) | 2-step immunoassay (I-module) |
|  | Vitamin D, nmol/L (50–150) | Immunoassay (I-module) |
|  | Albumin, g/L (36–45) | Colorimetry (C-module) |
|  | Total calcium, mmol/L (2.15–2.15) | Colorimetry (C-module) |
|  | Albumin corr calcium, mmol/L (2.17–2.47) | Calculated |
|  | Total cholesterol, mmol/L (3.3–6.9) | Enzymatic assay (C-module) |
|  | HDL cholesterol, mmol/L (0.8–2.1) | Enzymatic assay (C-module) |
|  | LDL cholesterol, mmol/L (1.5–4.8) | Enzymatic assay (C-module) |
|  | Triglycerides, mmol/L (0.45–2.60) | Enzymatic assay (C-module) |
| EDTA 6 ml | Biobanking plasma and cells |  |

Hb: hemoglobin, IgG: immunoglobulin G, HDL: high-density lipoprotein, LDL: low-density lipoprotein.

**Supplementary Table 2:**

WHO Trial Registration Data Set

| **Primary Registry and Trial Identifying Number** | ClinicalTrials.gov: NCT05179200 |
| --- | --- |
| **Date of Registration in Primary Registry** | December 20^th^, 2021 |
| **Secondary Identifying Numbers** | **Not applicable** |
| **Source(s) of Monetary or Material Support** | Department of Research, Innlandet Hospital Trust, Norway |
| **Primary Sponsor** | Department of Research, Innlandet Hospital Trust, Norway |
| **Secondary Sponsor(s)** | **Not applicable** |
| **Contact for Public Queries** | MH, [morten.haugen@sykehuset-innlandet.no](mailto:morten.haugen@sykehuset-innlandet.no), tel: +4790551681, Department of Immunology and Transfusion Medicine, Innlandet Hospital Trust, Anders Sandvigs gate 17, 2609 Lillehammer, Norway |
| **Contact for Scientific Queries** | TAS (Principal Investigator), [tors@me.com](mailto:tors@me.com)  Department of Research, Innlandet Hospital Trust, Norway |
| **Public Title** | Donor Health in Repeat Plasma Donors |
| **Scientific Title** | The Effect of Donation Frequency on Donor Health in Blood Donors Donating Plasma by Plasmapheresis: Randomized Controlled Trial |
| **Countries of Recruitment** | Norway |
| **Health Condition(s) or Problem(s) Studied** | Plasma protein deficiency, psychological distress |
| **Intervention(s)** | Active comparator:   - High-frequency plasma donors (HFPDs): Plasma donation of 650 mL by plasmapheresis 3 times every 2 weeks - Regular-frequency plasma donors (RFPDs): Plasma donation of 650 mL by plasmapheresis 1 time every 2 weeks   Control: Blood donors donating 450 mL of whole blood every 3 months |
| **Key Inclusion and Exclusion Criteria** | Age 18-65 years  Sex: Male  Established blood donors with ≥1 previous plasma donation  Hemoglobin ≥13.5 g/dL  Immunoglobulin G ≥6.0 g/L  Total serum protein ≥60 g/L  Estimated blood volume ≥4500 mL  Exclusion criteria: Repeatedly hematocrit >50% |
| **Study Type** | Interventional  Allocation: Randomized  Assignment: Two intervention arms  Purpose: Measure the effect of plasma donation frequency on donor health |
| **Date of First Enrollment** | January 2022 |
| **Sample Size** | 120 |
| **Recruitment Status** | Complete |
| **Primary Outcome(s)** | Change from baseline of total serum protein and immunoglobulin G concentrations (g/L) after the donation period (week 16) |
| **Key Secondary Outcomes** | Change from baseline of total serum protein and immunoglobulin G concentrations (g/L) at 10 time points (every 2 weeks) throughout the donation period |
| **Ethics Review** | Approved by Regional Committee for Medical and Health Research Ethics of South‒East Norway (2021/238929/REK Sør-Øst A)  Postal addressPostboks 1130 Blindern, 0318 Oslo, Norway |
| **Completion date** | July 2024 |
| **Summary Results** | Not applicable |
| **IPD sharing statement** | Plan to share IPHD: No |
