# Supplementary material for: The effect of donation frequency on donor health in blood donors donating plasma by plasmapheresis: study protocol for a randomized controlled trial
Source: Trials. 2024 Mar 11;25:175. doi: 10.1186/s13063-024-08035-7 (PMC10926559; doi:10.1186/s13063-024-08035-7)
Supplement: Supplementary file 2 — Additional file 2. Data and Safety Monitoring Board (DSMB) Charter. [file 13063_2024_8035_MOESM2_ESM.pdf]

DATA AND SAFETY MONITORING BOARD (DSMB) CHARTER

Confidential

**Trial:** Plasmapheresis project	**EUdraCT / REK no:** 238929
**Sponsor:** Innlandet Hospital Trust, Lillehammer, Norway	**Coordinating investigator:** Tor A Strand
**Date of Charter:** 23.03.2022	**Protocol:** Project description Plasma project dated 22.09.21

"The effect of donation frequency on plasma protein composition, inflammation markers and psychological distress in blood donors donating plasma by plasmapheresis – a randomized controlled trial"

Sponsor Signature page

"The effect of donation frequency on plasma protein composition, inflammation markers and psychological distress in blood donors donating plasma by plasmapheresis – a randomized controlled trial"

Version/date: 1.0/23.03.2022

Reviewed and Accepted by sponsor:

SPONSOR REPRESENTATIVE:

Name, title

[signature] 23/03/2022

Signature | Date (DD/MMM/YYYY)

1 STUDY OVERVIEW

Trial name: "The effect of donation frequency on plasma protein composition, inflammation markers and psychological distress in blood donors donating plasma by plasmapheresis – a randomized controlled trial"

Trial Sponsor and funding: Innlandet Hospital Trust, Lillehammer

Trial Design: Randomized controlled trial with control group

Number of subjects: 120 male voluntarily non-remunerated blood donors

Objectives of the trial: The objectives for this trial are, in a randomized controlled trial, to compare the current and previous guideline for plasmapheresis and study the effect of donation frequency on plasma protein composition, inflammation markers and psychological distress as parameters for donor health, to ensure safety of donors.

The primary objective is to compare the total serum protein (TSP) (g/L) and immunoglobulin G (IgG) (g/L) concentrations between donors who will be donating plasma 3 times every 2 weeks, donors donating plasma every 2 weeks, and donors who donate whole blood every 3 months. Secondary objectives are to compare the concentrations of other plasma proteins, biomarkers reflecting nutritional status and inflammation and to compare the dropout rate and reasons and the degree of psychological distress, between the three donation groups.

2 STATEMENT OF PURPOSE

2.1 Purpose of Data and Safety Monitoring board

The purpose of an independent Data and Safety Monitoring Board (DSMB) is to assess the progress of the trial, the safety data, and the critical efficacy endpoints and to provide recommendations to the sponsor. The members of the DSMB serve in an individual capacity and provide their expertise, including recommendations regarding the continuation, modification, or termination of any or all groups of the study. The DSMB will review cumulative study data to evaluate safety, study conduct, scientific validity and data integrity of the study.

2.2 Purpose of DSMB Charter

This charter describes the roles and responsibilities of the DSMB for the for the "Plasmapheresis project", delineates qualifications of the membership, including the timing of meetings, methods of providing information to and from the data monitoring committee (DMC), frequency and format of meetings and statistical issues. This

charter will serve as the Standard operating Procedure (SOP) for the DSMB. The DSMB will be independent of the sponsors and investigators.

3 DSMB MEMBERSHIP

- DSMB members will sign confidentiality agreements covering DSMB activities.
- Remuneration will be provided by the study sponsor in accordance with standard procedures of the DSMB.
- The Committee will be composed of three members (inclusive of the DSMB Chair). The DSMB includes experts in or representatives of the fields of immunology and transfusion medicine, and clinical trials methodology. The DSMB also includes at least one individual with competence on statistical methods for clinical research and analysis of trial data.
- Each DSMB member will be expected to serve for the duration of the trial; in the unlikely event that a member is unable to continue participation, the reason will be documented and a replacement will be selected by the sponsor.

4 COMPOSITION OF DSMB

The members of the DSMB are:

Name	Role	Expertise
Bjarte Erikstein	Chair	Immunology and transfusion medicine
Andreas Simensen	Member	Anaesthesiologist
Vegard Lysne	Member	Statistician

The DMC Chair will arrange meetings, facilitate and summarise discussions and approve any reports generated by the DMC.

The DMC statistician will receive and analyse data from the study team, following the same procedure as those described in the statistical analysis plan or protocol. The DMC statistician will interpret any statistical information provided by the study team.

5 INDEPENDENCE OF DSMB

It is essential that the judgment of members of the DSMB not will be influenced by factors other than those necessary to maintain subject safety, and to preserve the integrity of the study. Independence is essential to ensure that DSMB members are

objective and capable of an unbiased assessment of the study's safety and efficacy data. By agreeing to be a board member, the member is stating that there is no conflict of interest with regard to the trial under review by the DSMB.

Conflict of Interest
DSMB members should have no other relationship with the sponsor that could impair the members' ability to objectively review study data as set forth below:

- DSMB members must not have any real or perceived scientific, financial, professional, personal, proprietary, or other conflict of interest related to the conduct, outcome, or impact of the study. This may include having been or being employed by the sponsor/ investigator, having a fiduciary interest in the sponsor, conducting and/or managing the study, and/or having contact with participants during the course of regular clinical care;
- DSMB members must not be engaged in any simultaneously occurring competitive studies in any role that could pose a conflict of interest. DSMB members must also identify and disclose any concurrent service on other DSMBs of the same, related, or competing products;
- Collaborators or associates of the participating investigator(s) are not eligible to serve on the DSMB.

6 RESPONSIBILITIES OF THE DSMB

The following defines the DSMB responsibilities:

- To evaluate, on an ongoing basis, the accumulating safety assessments to ensure the ongoing safety of study subjects
- To review all documents provided in the DSMB data review packets upon receipt
- To review the conduct of the study, including protocol violations
- To review data on participant recruitment, accrual, and retention, as well as assessments of data quality, completeness, and timeliness
- Protect the confidentiality of the study data and the DSMB discussions
- To make recommendations to continue, modify, or terminate the study
- Operate according to the procedures described in this charter and all procedures of the DSMB
- Follow conflict of interest guidelines as detailed in this charter
- Maintain documentation and records of all activities as described below (see DSMB Meetings, DSMB Reports)

7 DSMB CHAIR RESPONSIBILITIES

The following responsibilities are those of the DSMB Chair:

- Serves as a voting member

- Facilitates the meetings, assists in the development of the agenda, and ensures that the meeting minutes and recommendation(s) are appropriately documented
- Serves as the primary contact person for the DSMB
- Reviews and approves the Charter
- Ensures that those involved in the day-to-day management of the study are excluded from DSMB voting procedures
- Discusses DSMB recommendations with appropriate members of the study team
- Takes and maintains minutes from DSMB sessions, or delegates another member to do so

8 MEETINGS OF THE DSMB

8.1 Communications

Members will be notified about meetings via email.

Projected Schedule of Meetings
An initial meeting of the DSMB will be held in the initial phase of the trial enrollment in order for the members to review the charter, form an understanding of the protocol and definitions being used, establish a distribution and meeting schedule, review the study modification and/or termination guidelines, and finalize format and protocol-specified statistical methods to be used in reports to be considered by the DSMB. Subsequent DSMB meetings will be held to review and discuss study data according to the schedule as described in the table below.

Timeline	Data Review By	Type of Data
Every 6 months	Chair	Adverse events, protocol violations, enrollment summary
When follow-up is completed on first 60 participants	Entire DSMB	Adverse events, protocol violations, enrollment summary
Upon completion or termination of study	Entire DSMB	Adverse events, protocol violations, enrollment summary

Ad Hoc Meetings
An ad hoc meeting of the DSMB may be called at any time by the DSMB Chair, investigator or sponsor, if imminent participant safety issues arise. If a significant safety concern arises during the study, the DSMB Chair may convene a meeting to review safety and any other aspect of the study.

Significant safety events may include, but are not limited to, the following:
• A death or life-threatening condition sustained by a participant, regardless of causality
• An unexpected serious safety issue newly identified during the development program that could expose participants to unnecessary risks
• Any other concern regarding participant safety raised by any DSMB member, investigator or sponsor.

Proposed study amendments that significantly alter the treatment plan and/or deal with participant safety concerns will prompt an ad hoc meeting of the DSMB for review prior to implementation of changes. This may require suspension of enrollment pending DSMB review.

8.2 Meeting Format

DSMB meetings will generally be conducted by teleconference/face-to-face meeting and facilitated by the DSMB Chair, consisting of an open session and a closed session.
A quorum, defined as at least 2 members, including the DSMB Chair must be present and is required to hold a DSMB meeting.

Open Session
The open session may be attended by the investigator(s) and representatives of the sponsor.The study statistician must always attend. Investigator and sponsor representatives may attend the open session with DSMB members, during which time they can provide information on, for example: recruitment updates, information on study conduct, compliance, withdrawals, data quality, and other blinded data and non-confidential information regarding operational/logistical issues. This session gives the DSMB an opportunity to query investigator about issues that have arisen during the review of safety data. Unblinded information will not be discussed in the open session.

Closed Session
The closed session will be restricted to attendance by the DSMB members. At the closed session, study blinding may be broken. Closed sessions also consist of review of the recommendations the DSMB wishes to make to the investigator and/or sponsor and a formal vote.

Voting
DSMB recommendations will be agreed upon by formal majority vote. In event of a split vote, the DSMB Chair will cast the deciding vote.

Stopping Rules

After considering the information in the open and closed session DSMB report, the DSMB will determine whether the study should continue as planned, proceed with modifications, or be terminated. The justification to terminate the study may be due to the DSMB's analysis that there is overwhelming effectiveness, futility, or safety issues. If the DSMB votes to terminate the study, the statistician will prepare a final study report for the DSMB and a final DSMB meeting will be held. The DSMB's recommendations at the final DSMB meeting may include continuing action items to sponsor, investigator(s) based upon the final review.

8.3 Materials

The primary charge of the DSMB is to monitor the study for participant safety. A feedback form for the DSMB containing relevant information will be prepared by the data management team in consultation with the project management team and the site investigators and sent to the DSMB at regular intervals.

The safety and related data the DSMB will review includes:
• Participant recruitment, accrual, retention, and withdrawal information
• Adverse events (AEs) and serious adverse events (SAEs)
• Any other safety-supporting data requested by the DSMB

Serious adverse events (SAEs) will be monitored by the DSMB Chair in real time throughout the study. SAEs must be reported by the investigator(s) to the sponsor and DSMB Chair via email in a PDF file, within one working day of learning of the event.

All participant withdrawals will be monitored by the DSMB Chair in real time throughout the study. All participant withdrawals must be reported by the investigator(s) to the sponsor and the DSMB Chair, via email in a PDF file, within one working day of learning of the withdrawal.

8.4 Minutes

A formal report of the meeting minutes containing recommendations for continuation or modification of the study will be prepared by the DSMB Chairperson or designee. A draft report will be sent to the DSMB members prior to distribution to the sponsor. DSMB members will have 5 days to review and respond to the draft report. The recommendations will then be sent to the sponsor. It is the responsibility of the sponsor to distribute recommendations to all investigators. The study team is responsible for informing REC if necessary.

9 RECOMMENDATIONS

The DSMB can recommend that the current study continue without modification, continue with specified modifications, discontinue one or more groups of the study, or halt or modify the study until more information is available.

10 STUDY REVIEW CRITERIA/STOPPING RULES AND GUIDELINES

The DSMB may recommend stopping the study for the following reasons:

- The data show a significantly increased risk of serious adverse effects in one of the treatment groups.
- It becomes clear that successful completion of the study is not feasible (e.g. there is an excess of patient dropout, missing data, lack of recruitment etc).

If the DSMB votes to terminate the study, the DSMB chair will prepare a final study report for the DSMB and a final DSMB meeting will be held.

11 AMENDEMENTS TO THE CHARTER

This DSMB charter can be amended as needed during the course of the study. Information to be included as amendments will be any modifications or supplements to the reports prepared for the DSMB, as well as amendments to other information addressed in this charter. All amendments will be documented with sequential version numbers and revision dates, and will be recorded in the minutes of the DSMB meetings. All versions of the charter will be archived in accordance with this document.

12 COMPLETION OF DSMB ACTIVITIES

The activities of the DSMB will be considered when all sites have completed enrollment and a final review of SAEs has taken place.

13 CONFIDENTIALITY

All data provided to the DSMB and all deliberations of the DSMB will be privileged and confidential. The DSMB will agree to use this information to accomplish the responsibilities of the DSMB and will not use it for other purposes without written consent from the study sponsor as specified in this document. Individual DSMB members must not have direct communication regarding the study outside the DSMB (including, but not limited to the investigators, IRB/ethics committees (EC), regulatory agencies, or sponsor) except as authorized by the DSMB.

Data Monitoring Committee members for

**Protocol**	Project description Plasma project dated 22.09.21
**Sponsor**	Innlandet Hospital Trust, Lillehammer, Norway

DSMB Member Signature Page

Member Information

Role: DSMB Chair _____ Member ____

Voting Rights: Yes ____ No ___

Name:
Affiliation:
Phone:
Fax:
E-mail address:

Re: DSMB Charter Version Date: _____________

I have reviewed the attached DSMB Charter and approve it as written. I understand my role as a member of this DSMB, and will adhere to the confidentiality and conflict of interest policies, as stated in this document.

Signature Date (DD/MMM/YYYY)

14 ATTACHMENT (NOT INCLUDED IN PROTOCOL)

14.1 Discontinuation criteria

Criteria that leads to temporary discontinuation of study:

1. IgG levels:
    a. Two subsequent measurements of IgG<6.0 g/L, leads to break in donations of at least two weeks. If new IgG>6.0, the donor may continue donations.
2. TSP levels:
    a. Two subsequent measurements of TSP<60 g/L, leads to break in donations of at least two weeks. If new TSP>60 g/L, the donor may continue donations.
3. Haemoglobin level:
    a. Haemoglobin <13.5 (blood donor)/<13.0 (plasma donor) g/dL leads to break in donations of at least 2 weeks*. If new haemoglobin >13.5/13.0 g/dL measured later, the donor may continue donations.
4. Failed return of red cells during a plasmapheresis
    a. Leads to break in donations of at least one month (assessed by medical doctor at the blood centre)
5. Adverse events grade 1-2
    a. May lead to break in donations and will be assessed individually

If the donor fail to return for more than one following plasma donation in donation group I or II, this leads to discontinuation of per-protocol analyses. These may still be included in the intent to treat group (if not permanent discontinuation). In addition, at least 90% of the donations are needed to be included in the per-protocol analyses.
*Donors will be given iron supplement according to local procedures if haemoglobin or ferritin levels are low.

Criteria that leads to permanent discontinuation of study:

1. Adverse events grade 3-5
2. The blood donor asks to be withdrawn from the study
3. Lost to follow-up: Donors repeatedly fails to return for scheduled visits and is unable to be contacted
4. Medical reasons

14.2 Categorization of adverse events by severity

Grade of severity will be evaluated if adverse events.

1. Grade 1: Medical intervention not necessary
2. Grade 2: Monitoring and minimum medical intervention

3. Grade 3: Major medical intervention or hospitalization
4. Grade 4: Life-threatening
5. Grade 5: Lethal

Relation to plasmapheresis of each adverse event to plasmapheresis will be evaluated:

1. No relation
2. Unexpected side effect
3. Cannot be ruled out
4. Probable or certain

14.3 Dropout categories

Blood and plasma donors will be asked about reason for dropout if they voluntarily want to end the study. Reasons for dropout will be categorized as following:

1. Medical reasons related to plasmapheresis or blood donations
    a. Low TSP (<60 g/L)
    b. Low S-IgG (<6.0 g/L)
    c. Low Hb (<12.5 g/dL for plasma donations, <13.5 g/dL for Hb blood donations)
    d. Others: Venipuncture related, citrate reactions, side effects – dizziness, nausea, vomiting, hypotensive
2. Medical reasons not related to plasmapheresis or blood donations
    a. Medical diseases
    b. Surgery, accidents, injuries
    c. General discomfort
    d. Investigation of suspect disease, diagnostics
    e. Blood test findings not related to plasmapheresis
        i. Pathological hematologic, microbiologic or biochemical blood tests
3. Socioeconomic reasons
    a. Lack of time, schedule conflicts
    b. Moving out of area
    c. Unsatisfactory compensation, transport problems, discomfort, concerns about health, piercing, tattoo etc.
    d. Others
